# Preoperative continuation vs. discontinuation of angiotensin-converting enzyme inhibitors/angiotensin II receptor blockers on early cognitive function in elderly patients undergoing noncardiac surgery: a randomized controlled trial

**DOI:** 10.3389/fnagi.2025.1542628

**Published:** 2025-03-17

**Authors:** Xiaohan Wang, Yanan Yan, Yurong Liu, Chun Xu, Jingwen Zhuang, Zhiping Wang

**Affiliations:** ^1^School of Anesthesiology, Xuzhou Medical University, Xuzhou, Jiangsu, China; ^2^Jiangsu Province Key Laboratory of Anesthesiology, Xuzhou Medical University, Xuzhou, Jiangsu, China; ^3^Jiangsu Province Key Laboratory of Anesthesia and Analgesia Application Technology, Xuzhou Medical University, Xuzhou, Jiangsu, China; ^4^National Medical Products Administration (NMPA) Key Laboratory for Research and Evaluation of Narcotic and Psychotropic Drugs, Xuzhou Medical University, Xuzhou, Jiangsu, China; ^5^Department of Anesthesiology, The Affiliated Hospital of Xuzhou Medical University, Xuzhou, Jiangsu, China

**Keywords:** angiotensin-converting enzyme inhibitors, angiotensin II receptor blockers, cognitive function, elderly, hypotension

## Abstract

**Objectives:**

To evaluate the effect of preoperative continuation vs. discontinuation of angiotensin-converting enzyme inhibitors (ACEIs) and angiotensin II receptor blockers (ARBs) on early cognitive function in elderly patients undergoing noncardiac surgery.

**Methods:**

This prospective randomized controlled study was performed at the Affiliated Hospital of Xuzhou Medical University. Elderly patients aged 65 years or older, scheduled for elective noncardiac surgery under general anesthesia, and receiving long-term ACEI/ARBs therapy were randomly assigned to either continue or discontinue ACEI/ARBs therapy on the morning of surgery. The primary outcome was postoperative early cognitive function, assessed via neuropsychological tests including Auditory Verbal Learning Test-Huashan (AVLT-H), Clock Drawing Test (CDT), Number Connection Test (NCT), and Digit Span Test (DST) preoperatively and on postoperative day 1 (POD1). Secondary outcomes included intraoperative hypotension, use of phenylephrine, intraoperative fluid administration, incidence of hypertension, and length of hospital stay.

**Results:**

The NCT scores in the discontinued use of ACEI/ARBs group showed a significant decline on POD1 compared to baseline (*p* = 0.038). Both groups exhibited an increase in immediate recall scores from preoperative to POD1 (*p* = 0.003 and *p* = 0.002, respectively). The continued use of ACEI/ARBs group showed an increase in short-delayed recall (*p* = 0.007). However, there were no significant differences between the two groups (*p* > 0.05). The discontinued ACEI/ARB group had fewer episodes of intraoperative hypotension (*p* = 0.037) and lower requirements for phenylephrine (*p* = 0.016), despite a higher incidence of preoperative hypertension (*p* = 0.012). The continued use group received a larger volume of crystalloid fluids during surgery (*p* = 0.020). No significant differences were observed between the groups in the volume of colloid fluids administered (*p* > 0.05). There were no significant differences in postoperative hypertension or length of hospital stay between the groups (*p* > 0.05).

**Conclusion:**

Preoperative continuation or discontinuation of ACEI/ARBs did not significantly affect early postoperative cognitive function in elderly patients.

## 1 Introduction

Angiotensin-converting enzyme inhibitors (ACEIs) and angiotensin II receptor blockers (ARBs) are frequently used in the treatment of hypertension and heart failure (Halvorsen et al., 2022), especially in elderly patients. These medications regulate blood pressure and fluid balance by inhibiting the renin-angiotensin-aldosterone system (RAAS) (Ksiazek et al., 2024). However, their use in elderly patients undergoing surgery has raised concerns about their potential impact on cognitive function (Halvorsen et al., 2024; Legrand, 2024). Postoperative cognitive dysfunction (POCD) is a common complication observed in older adults, associated with increased morbidity, longer hospital stays, and a higher risk of mortality (Bedford, 1955; Steinmetz and Rasmussen, 2016). POCD is typically diagnosed through neuropsychological testing (Moller et al., 1998), but its underlying mechanisms remain unclear. Possible contributing factors include neuroinflammation (Hovens et al., 2014), cerebral microembolism (Liu et al., 2009), and undetected perioperative stroke (Mrkobrada et al., 2019). As the number of elderly patients undergoing noncardiac surgery continues to rise, understanding the effects of ACEIs and ARBs on cognitive outcomes is crucial for optimizing perioperative care.

Several studies have examined the relationship between ACEIs/ARBs and cognitive function, yielding inconsistent results. Some suggest that inhibiting the RAAS may protect against cognitive decline by enhancing the memory-protective effects of the angiotensin type 4 receptor (AT4R) (Cosarderelioglu et al., 2023), improving cerebral perfusion, and reducing oxidative stress. However, other research indicates that continuing these medications before surgery may increase the risk of intraoperative hypotension (IOH) (Hollmann et al., 2018), which can impair cerebral perfusion (Krenk et al., 2010; Pappa et al., 2017). Cerebral autoregulation, a mechanism that stabilizes blood flow to the brain (Armstead, 2016), is disrupted during IOH, leading to hypoperfusion and potential ischemic injury. This can disturb brain homeostasis and contribute to postoperative cognitive dysfunction (POCD) (Wang, 2014). The conflicting findings highlight the need for further investigation into how ACEIs and ARBs affect postoperative cognitive outcomes, particularly in elderly patients who are particularly vulnerable to both surgical stress and polypharmacy.

The objective of this study is to investigate the impact of continuing vs. discontinuing ACEIs/ARBs on cognitive function in elderly patients undergoing elective noncardiac surgery. We hypothesize that continued use of these medications may impair cognitive outcomes, potentially due to their impact on blood pressure regulation. Conversely, discontinuing ACEIs/ARBs prior to surgery may reduce the risk of hypotension and its negative effects on cerebral blood flow, thereby improving cognitive outcomes. This randomized controlled trial seeks to provide clearer evidence on the role of these medications in postoperative early cognitive recovery, ultimately guiding clinical decision-making in perioperative care.

## 2 Methods

### 2.1 Study design

This prospective, randomized controlled trial was carried out at the Affiliated Hospital of Xuzhou Medical University, a 4150-bed tertiary university-affiliated teaching hospital in Jiangsu, China. The trial protocol received approval from the hospital's medical ethics committee (Approval Number: XYFY2024-KL529-01) and was registered with the Chinese Clinical Trials Registry (Registration Number: ChiCTR2400091763). The study adhered to the Consolidated Standards of Reporting Trials (CONSORT) guidelines. Written informed consent was obtained from all participants before their enrollment in the study.

### 2.2 Population selection

Patients aged 65 years or older were eligible for inclusion if they were scheduled for elective noncardiac surgery under general anesthesia and had been on long-term treatment with ACEIs or ARBs for a minimum of three months prior to the surgery. These medications were taken once daily in the morning.

The exclusion criteria included pre-existing cognitive impairment assessed by Montreal Cognitive Assessment (MoCA) (scores ≤ 17 for illiterate, ≤ 20 for primary, ≤ 22 for junior high, and ≤ 24 for university), inadequate blood pressure control with antihypertensive medications, preoperative hypotension (blood pressure < 90/60 mmHg), mental or neurological disorders or history of stroke, use of neuroprotective drugs, surgeries requiring controlled hypotension (e.g., hepatectomy, rotator cuff repair), and impairments in speech, vision, or hearing.

The rejection criteria included patients diagnosed with delirium using the Confusion Assessment Method (CAM) scale one day after surgery.

### 2.3 Randomization, blinding, and intervention

Eligible patients were randomly assigned to either continue or discontinue ACEI/ARBs in a 1:1 ratio, using a computer-generated randomization system. An investigator, who was not involved in data collection, informed patients of their group assignment. A second investigator, blinded to the group assignments, recorded and assessed the data. In the continuation group, ACEI/ARBs therapy was maintained on the morning of surgery. In the discontinuation group, ACEI/ARBs therapy was stopped on the morning of surgery, with the last dose taken 1 day prior. The night before surgery, a ward nurse informed patients whether to continue or discontinue their medication. Before entering the operating room, the nurse confirmed that patients had taken their antihypertensive medications as instructed. Patients who did not adhere to the medication instructions were excluded from the trial.

### 2.4 Anesthesia protocol

Anesthetic management was standardized based on current best practices by experienced anesthesiologists, who were blinded to the patients' preoperative ACEI/ARBs continuation/discontinuation status. After the patient entered the operating room, the venous access to the elbow of the right upper limb was opened. Non-invasive blood pressure, electrocardiogram (ECG), saturation of pulse oximetry (SpO_2_), and bispect ral index (BIS) were routinely monitored. After local anesthesia with lidocaine, invasive blood pressure was monitored by puncture and catheterization of the radial artery under ultrasound guidance.

All patients received intravenous induction with the same drugs. General anesthesia was induced by intravenous injection of midazolam 0.05 mg/kg, sufentanil 0.5 μg/kg, etomidate 0.3 mg/kg and rocuronium 0.6 mg/kg. When BIS dropped below 60, endotracheal intubation was performed. The intubation depth was 23 cm. The endotracheal tube was connected to the breathing circuit and the volume control mode was used for mechanical ventilation. Low tidal volume was 6–8 mL/kg, respiratory rate was 12–20 times/min, inspiratory/expiratory ratio was 1:1.5, oxygen flow rate was 2 L/min, and end-tidal carbon dioxide partial pressure was maintained at 35–45 mmHg. Anesthesia was maintained with combined intravenous and inhalant anesthesia, 1–2% sevoflurane was inhaled, 0.2–0.5 μg/kg/min remifentanil and 2–6 mg/kg/h propofol were pumped intravenously, and BIS value was maintained at 40–60. If the heart rate was < 50 beats per min, atropine 0.25–0.50 mg was administered intravenously, and the dose could be repeated if necessary. Multi-mode temperature protection measures and nasopharyngeal temperature monitoring were adopted during the whole operation. Postoperative pain was controlled with an intravenous injection of 50 mg flurbiprofen axetil at the time the surgeon started the skin suture.

After the operation, the patient was transferred to the postanesthesia care unit (PACU) for close monitoring. The patient's degree of wakefulness, the smoothness of spontaneous breathing, the presence of swallowing reflexes, and the recovery of muscle strength were carefully assessed. The tube was extubated when the tidal volume was >6 mL/kg. After extubation, the patient still needs to continue to be observed in the PACU for a period of time to ensure that vital signs are stable and there are no adverse effects.

### 2.5 Data collection and study outcomes

Baseline patient characteristics collected included age, sex, BMI, education level, current smoking status, comorbidities (diabetes mellitus, coronary artery disease, cancer, and history of general anesthesia), preoperative laboratory values (hemoglobin and creatinine), preoperative hemodynamic parameters (blood pressure and pulse), and preoperative cognitive assessment. Intraoperative data extracted included the type and duration of surgery, as well as the need for transfusion.

The primary outcome was postoperative early cognitive function, assessed using neuropsychological tests on postoperative day 1 (POD1). Secondary outcomes included the incidence of hypotension within 15 min of anesthesia induction (defined as a 30% decrease in systolic blood pressure from baseline or a mean arterial pressure < 55 mmHg), intraoperative phenylephrine dosage (μg/h), infusion volume (crystalloid and colloid fluids), incidence of hypertension upon admission to the operating room and within 24 h post-surgery, and length of hospital stay.

### 2.6 Neuropsychological test*s*

To maintain standardization and consistency in data collection, widely recognized neuropsychological tests were employed (Jannati et al., 2024; Stefanidis et al., 2025).

The Auditory Verbal Learning Test-Huashan (AVLT-H) involves the researcher reading 12 words aloud, and the patient is asked to recall as many words as possible immediately afterward. The total number of words recalled across three trials is recorded as the immediate recall score, which assesses the patient's capacity to learn new information. A short-delayed recall test is administered after a 5-min interval, and a long-delayed recall test is performed after 20 min. The short-delayed and long-delayed recall scores reflect the patient's ability to retain new information over time. Higher scores on the AVLT-H indicate better memory function.

The Clock Drawing Test (CDT) requires the patient to draw a clock on a blank piece of paper, ensuring all numbers are included. The hour and minute hands should indicate 11:10. Scoring is based on the Rouleau scale, which evaluates three components: completeness of the clock face (maximum 2 scores), correct display and order of numbers (maximum 4 scores), and proper positioning of the hands (maximum 4 scores). The CDT is a screening tool used to assess cognitive function and detect early signs of dementia. A lower total CDT score indicates worse cognitive function.

The Number Connection Test (NCT) requires the patient to connect 25 randomly arranged circles, numbered from 1 to 25, in order from smallest to largest. The time taken to complete the task is recorded. The total score reflects executive function, with a lower score indicating better executive function.

The Digit Span Test (DST) requires the patient to recite a series of numbers in either sequential or reverse order. The total score is based on the longest sequence of digits the patient can accurately recall. The DST measures memory span, with a higher score indicating a greater memory span.

### 2.7 Statistical analysis

Data analysis was conducted using SPSS version 25.0. Normally distributed measurement data are presented as mean ± standard deviation, while non-normally distributed data are presented as median (M) and interquartile range (IQR). Independent sample *t*-tests were employed to compare normally distributed data between the two groups, and the Mann-Whitney *U* test was used for non-normally distributed data. Categorical data are presented as counts (percentages), and group comparisons were made using the chi-square test or Fisher's exact test, as appropriate. A *p*-value of < 0.05 was considered statistically significant.

The immediate recall score of the AVLT-H was used as the primary endpoint for sample size calculation. Drawing from the results of a pilot study, the immediate recall score in the continued use of ACEI/ARBs group was 13.93 ± 4.01, and in the discontinued use of ACEI/ARBs group, it was 15.80 ± 3.49. With an alpha level set at 0.05 and a power of 0.80, the calculated sample size required for each group was 51. To account for a 10% dropout rate, 57 patients per group were needed, resulting in a total sample size of 114.

## 3 Results

### 3.1 Baseline characteristics of the patients and details of surgery

**Figure 1:**
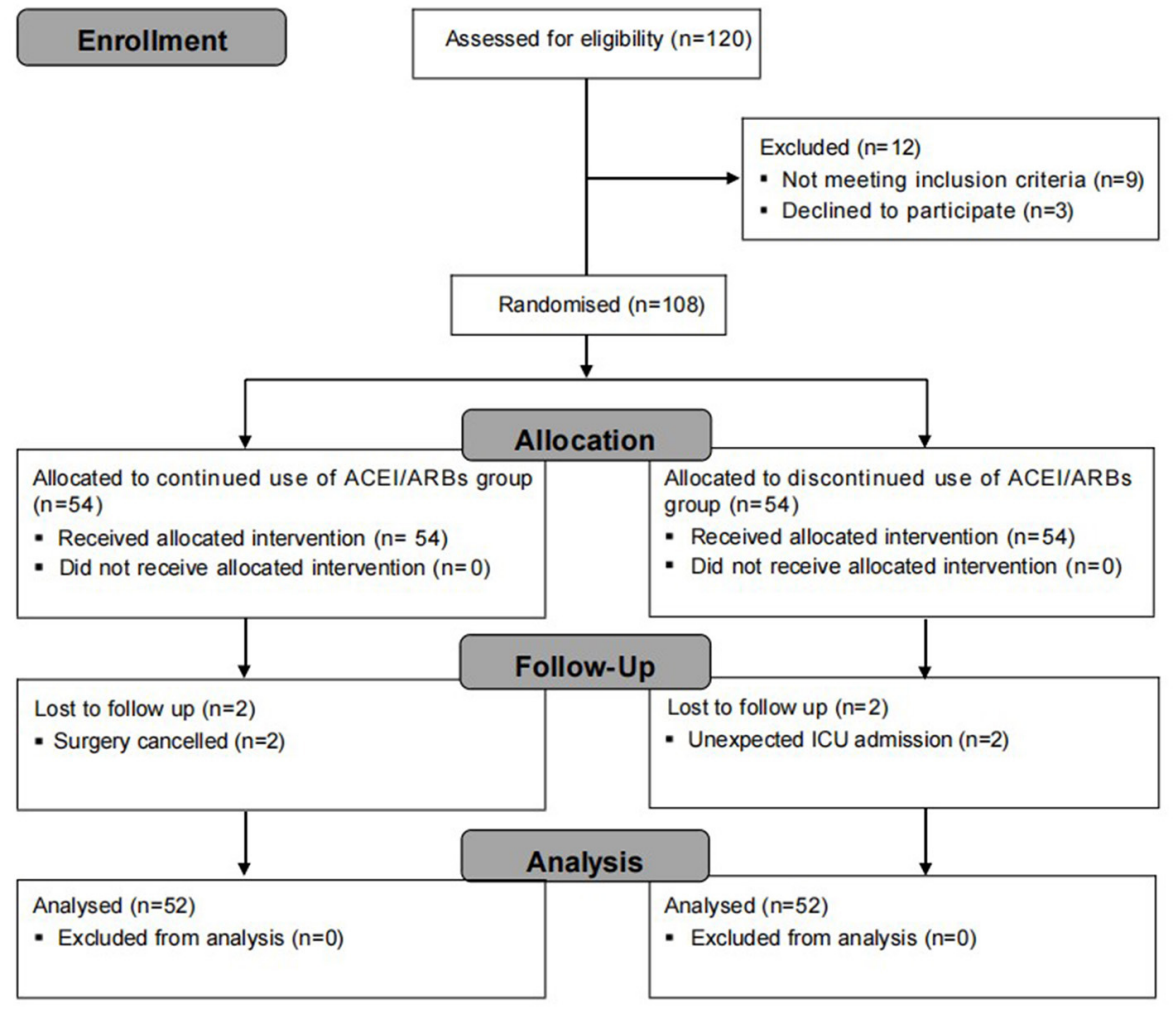
Flow chart of patient enrollment in this study.

Between September 2024 and November 2024, a total of 120 patients were screened for eligibility. Nine patients were excluded due to the exclusion criteria, and three patients opted not to participate in the study. As a result, 108 patients were recruited and randomly assigned to either the continued use of ACEI/ARBs group (*n* = 54) or the discontinued use of ACEI/ARBs group (*n* = 54). Four patients were lost to follow-up due to surgery cancellation or unexpected ICU admission. Ultimately, 104 patients completed the neuropsychological test on POD1 and were included in the analysis of the primary outcome (Figure 1). No significant differences were observed in baseline characteristics, surgical details or preoperative cognitive assessment between the two groups (Table 1).

**Table 1:** Baseline characteristics of the patients and details of surgery.

**Characteristics**	**Continued use of ACEI/ARBs (*n* = 52)**	**Discontinued use of ACEI/ARBs (*n* = 52)**	***p-*value**
Age, years	71.50 (68.00, 75.00)	71.00 (68.25, 75.00)	0.737
Male sex, *n* (%)	28 (53.8)	28 (53.8)	1.000
BMI, kg/m^2^	24.65 (3.46)	23.82 (2.90)	0.192
Education degree, *n* (%)			0.051
Primary school and below	27 (51.9)	26 (50.0)	
Junior high school	9 (17.3)	13 (25.0)	
Senior high school	8 (15.4)	12 (23.1)	
University diploma and above	8 (15.4)	1 (1.9)	
Antihypertensive medication category, *n* (%)			0.750
ACEIs use	5 (9.6)	6 (11.5)	
ARBs use	47 (90.4)	46 (88.5)	
**Comorbidities**, ***n*** **(%)**			
Diabetes	6 (11.5)	9 (17.3)	0.402
Coronary artery disease	10 (19.2)	6 (11.5)	0.277
Cancer	28 (25.5)	23 (44.2)	0.327
Currently smoke, *n* (%)	4 (7.7)	6 (11.5)	0.506
History of general anesthesia, *n* (%)	35 (67.3)	34 (65.4)	0.836
**Preoperative levels**			
Hemoglobin, g/L	131.04 (13.11)	126.04 (17.97)	0.108
Creatinine, mg/dL	59.50 (50.00, 66.75)	56.00 (49.00, 74.75)	0.930
**Preoperative hemodynamic parameters**			
Systolic blood pressure, mmHg	141.87 (17.08)	137.21 (17.43)	0.172
Diastolic blood pressure, mmHg	84.96 (11.30)	81.12 (9.92)	0.068
Pulse, bpm	78.00 (72.00, 84.75)	74.50 (70.25, 85.25)	0.415
Type of surgery, *n* (%)			0.510
Abdominal	18 (34.6)	16 (26.9)	
Thoracic	4 (7.7)	7 (13.5)	
Orthopedic	20 (38.5)	14 (26.9)	
Urological	5 (9.6)	8 (15.4)	
Gynecological	2 (3.8)	4 (7.7)	
Neurosurgical	3 (5.8)	5 (9.6)	
Duration of surgery, min	132.50 (86.25, 183.75)	100.00 (72.00, 180.00)	0.171
Blood transfusion, *n* (%)	2 (3.8)	0 (0.0)	0.475
**Preoperative cognitive assessment**			
AVLT-H: Immediate recall	12.00 (8.00, 16.00)	12.00 (8.00, 15.00)	0.860
AVLT-H: Short-delayed recall	4.00 (3.00, 5.00)	4.00 (3.00, 6.00)	0.457
AVLT-H: Long-delayed recall	3.00 (2.25, 4.75)	3.00 (2.00, 4.00)	0.585
CDT	9.00 (8.00, 10.00)	9.00 (7.00, 10.00)	0.755
NCT	73.50 (54.00, 126.00)	80.00 (67.25, 120.00)	0.160
DST	8.00 (7.00, 9.00)	8.00 (7.00, 9.00)	0.645

ACEI, angiotensin-converting enzyme inhibitor; ARB, angiotensin II receptor blocker; ASA, American Society of Anesthesiologists; BMI, body mass index; AVLT-H, Auditory Verbal Learning Test-Huashan; CDT, Clock Drawing Test; NCT, Number Connection Test; DST, Digit Span Test.

Values are expressed as mean (standard deviation), median (interquartile range), or number (%).

### 3.2 Primary outcome

Cognitive function, as assessed by neuropsychological tests, is summarized in Table 2. The NCT scores in the discontinued use of ACEI/ARBs group showed a significant decline on POD1 compared to baseline (*p* = 0.038). In contrast, both groups exhibited an increase in immediate recall scores from preoperative to POD1 (*p* = 0.003 and *p* = 0.002, respectively). The continued use of ACEI/ARBs group also showed an improvement in short-delayed recall (*p* = 0.007). However, no significant changes were observed in other cognitive measures, including long-delayed recall, CDT, and DST, within either group (*p* > 0.05).

**Table 2:** Cognitive function assessed by neuropsychological tests.

**Variables**	**Continued use of ACEI/ARBs (*****n*** = **52)**			**Discontinued use of ACEI/ARBs (*****n*** = **52)**		

	**Preoperative**	**POD1**	***p-*** **value**	**Preoperative**	**POD1**	***p-*** **value**
AVLT-H: Immediate recall	12.00 (8.00, 16.00)	16.00 (12.00, 18.00)	0.003	12.00 (8.00, 15.00)	15.00 (11.00, 18.00)	0.002
AVLT-H: Short-delayed recall	4.00 (3.00, 5.00)	5.00 (4.00, 6.00)	0.007	4.00 (3.00, 6.00)	5.00 (3.00, 6.00)	0.214
AVLT-H: Long-delayed recall	3.00 (2.25, 4.75)	4.00 (3.00, 5.00)	0.249	3.00 (2.00, 4.00)	3.50 (2.00, 5.00)	0.389
CDT	9.00 (8.00, 10.00)	10.00 (9.00, 10.00)	0.087	9.00 (7.00, 10.00)	10.00 (8.00, 10.00)	0.392
NCT	73.50 (54.00, 126.00)	59.00 (47.00, 112.50)	0.073	80.00 (67.25, 120.00)	70.00 (51.00, 111.25)	0.038
DST	8.00 (7.00, 9.00)	8.50 (7.00, 9.00)	0.618	8.00 (7.00, 9.00)	8.00 (8.00, 9.00)	0.138

ACEI, angiotensin-converting enzyme inhibitor; ARB, angiotensin II receptor blocker; POD1, postoperative day 1; AVLT-H, Auditory Verbal Learning Test-Huashan; CDT, Clock Drawing Test; NCT, Number Connection Test; DST, Digit Span Test.

Values are expressed as median (interquartile range).

The two groups showed no significant differences in the magnitude of decline in cognitive function, as assessed by immediate recall, short-delayed recall, long-delayed recall, CDT, NCT, and DST (Table 3).

**Table 3:** Change in cognitive function from baseline.

**Variables**	**Continued use of ACEI/ARBs (*n* = 52)**	**Discontinued use of ACEI/ARBs (*n* = 52)**	***p-*value**
AVLT-H: Immediate recall	2.00 (0.00, 5.75)	2.50 (0.00, 5.00)	0.814
AVLT-H: Short-delayed recall	1.00 (0.00, 2.00)	0.00 (0.00, 1.00)	0.052
AVLT-H: Long-delayed recall	0.00 (0.00, 1.00)	0.00 (−1.00, 1.00)	0.414
CDT	0.00 (0.00, 1.00)	0.00 (0.00, 1.00)	0.338
NCT	−4.50 (−19.00, 3.00)	−5.00 (−23.00, 7.00)	0.805
DST	0.00 (0.00, 1.00)	0.00 (0.00, 1.00)	0.234

ACEI, angiotensin-converting enzyme inhibitor; ARB, angiotensin II receptor blocker; AVLT-H, Auditory Verbal Learning Test-Huashan; CDT, Clock Drawing Test; NCT, Number Connection Test; DST, Digit Span Test.

Values are expressed as median (interquartile range).

### 3.3 Secondary outcomes

The continued use of ACEI/ARBs group had a higher incidence of hypotension (42.3 vs. 23.1%, *p* = 0.037) and required a greater dose of phenylephrine per hour (3.73 μg/h vs. 1.29 μg/h, *p* = 0.016). Additionally, the continued use group received a larger volume of crystalloid fluids during surgery (1,450 vs. 1,200 mL, *p* = 0.020). However, no significant differences were observed between the groups in the volume of colloid fluids administered (*p* > 0.05). Regarding preoperative hypertension, the continued use group had a lower incidence (13.5 vs. 34.6%, *p* = 0.012). There were no significant differences in postoperative hypertension or length of hospital stay between the groups (Table 4).

**Table 4:** Secondary outcomes.

**Variables**	**Continued use of ACEI/ARBs (*n* = 52)**	**Discontinued use of ACEI/ARBs (*n* = 52)**	***p-*value**
Incidence of hypotension, *n* (%)	22 (42.3)	12 (23.1)	0.037
Phenylephrine per hour, ug/h	3.73 (0.00, 5.33)	1.29 (0.00, 4.00)	0.016
**Intraoperative infusion volume, mL**			
Crystalloid liquid	1,450.00 (1,025.00, 1,800.00)	1,200.00 (1,000.00, 1,500.00)	0.020
Colloid liquid	500.00 (0.00, 500.00)	0.00 (0.00, 500.00)	0.315
Preoperative hypertension, *n* (%)	7 (13.5)	18 (34.6)	0.012
Postoperative hypertension, *n* (%)	10 (19.2)	11 (21.2)	0.807
Length of hospital stay, days	10.00 (8.00, 13.00)	7.00 (7.00, 13.50)	0.104

ACEI, angiotensin-converting enzyme inhibitor; ARB, angiotensin II receptor blocker.

Values are expressed as median (interquartile range), or number (%).

## 4 Discussion

The primary findings of this study suggested that both the continuation and discontinuation of ACEI/ARBs before noncardiac surgery resulted in similar cognitive outcomes, as assessed by neuropsychological tests. Both groups showed some improvements or impairments in cognitive performance, but these changes were comparable between the groups. Patients in the ACEI/ARBs discontinuation group experienced fewer episodes of intraoperative hypotension and required less phenylephrine, despite a higher incidence of preoperative hypertension. These results indicated that while discontinuing ACEI/ARBs may offer advantages in blood pressure management during surgery, it did not have a significant impact on early cognitive function compared to continuing the medications.

Our findings are consistent with some previous studies but differ in certain respects. For example, a study by Erlandson et al. (2017) found modest declines in neurocognitive performance in specific domains associated with ACEI/ARB therapy, but no consistent evidence that these medications affected global neurocognitive function. Both studies suggested that while cognitive impairments may be observed postoperatively in certain areas—such as executive function, processing speed, or verbal learning and memory—no significant differences in cognitive outcomes were observed between patients who continued or discontinued ACEI/ARB therapy.

However, in contrast to our study, a systematic review (Stuhec et al., 2017) of 15 RCTs found that ARBs may improve cognitive function in elderly patients without prior cerebrovascular disease, particularly with regard to episodic memory. ACEIs, while similarly effective in lowering blood pressure, did not appear to improve cognitive function in the elderly. These findings align with a previous network meta-analysis (Levi Marpillat et al., 2013). On the other hand, a combined meta-analysis of RCTs and observational studies (Zhuang et al., 2016) suggested that centrally acting ACEIs (CACEIs) may help prevent cognitive decline, whereas ARBs showed no such benefit. The discrepancies between studies could be due to differences in antihypertensive drug classes, the specific cognitive tests used, or perioperative factors such as intraoperative blood pressure management, which appeared to influence outcomes in our cohort. Additional research is required to better understand the effects of CACEIs, peripheral ACEIs (PACEIs), and ARBs on cognitive function.

The mechanisms through which ACEIs and ARBs may affect cognitive function are complex and multifactorial. Emerging evidence suggests that both the peripheral and central nervous systems' renin-angiotensin system (RAS) (de Miranda et al., 2024), particularly the ACE/Ang II/angiotensin II type 1 receptor (AT1R) pathway (Leong et al., 2002), play crucial roles in regulating immune responses (Bauer and Teixeira, 2021), oxidative stress, and the activation of the hypothalamic-pituitary-adrenal (HPA) axis (Belvederi Murri et al., 2016). This pathway is implicated in neuroinflammation and cognitive decline, contributing to behavioral disorders and amyloid deposition, which further exacerbate cognitive impairment (Tiwari et al., 2023). Furthermore, the difference in protective effectiveness between ARBs and ACEIs, as mentioned before, may be attributed to their diverse mechanisms of antagonism toward independent receptor pathways or their disparate impacts on amyloid metabolism.

A deeper understanding of the cellular and molecular mechanisms underlying cognitive decline could lead to the identification of novel biological targets for therapeutic intervention. These insights might also pave the way for repurposing existing medications, such as RAS modulators, for cognitive-related disorders. Scotti et al.'s (2021) meta-analysis demonstrated that the employment of AT1R blockers contributed to a significant relieve of the risk of any dementia. Therefore, inhibiting the AT1R function (Vasconcelos et al., 2021) in the brain could present a promising therapeutic strategy for treating neuropsychiatric conditions linked to altered brain immune responses and cognitive dysfunction. However, clinical trials are essential to evaluate the efficacy of these potential treatments.

Our study found that patients in the continued ACEI/ARBs group experienced a higher incidence of intraoperative hypotension and greater use of vasoactive drugs, which aligns with findings from the broader literature. For example, a multicenter randomized clinical trial (Legrand et al., 2024) involving 2,222 patients undergoing major noncardiac surgery showed that those who continued renin-angiotensin system inhibitors (RASI) preoperatively had more frequent and prolonged episodes of intraoperative hypotension. Similar results were observed in ambulatory surgery patients (Gurunathan et al., 2024), where antihypertensive use was linked to a higher frequency of early and overall hypotension compared to those not on antihypertensive medications. This finding is further supported by a meta-analysis (Ahmed et al., 2024), which concluded that withholding RAAS inhibitors before noncardiac surgery significantly reduced intraoperative hypotension and the incidence of acute kidney injury (AKI) without impacting mortality or major adverse cardiovascular events (MACE). Intraoperative hypotension (IOH) is a common complication during noncardiac surgery (Ackland and Abbott, 2022), and it is associated with severe postoperative outcomes, such as 30-day mortality, AKI, and stroke (Cai et al., 2023). Our finding of increased preoperative hypertension with ACEI/ARB discontinuation before surgery also mirrors results from the SPACE trial by Ackland et al. (2024), which reported that discontinuing RAS inhibitors in noncardiac surgery patients raised the risk of clinically significant hypertensive events. Differences across studies may arise due to variations in patient demographics, surgical types, and institutional protocols. Future studies are needed to determine the optimal medication strategy that balances the prevention of both hypotension and hypertensive events.

Several limitations in our study must be acknowledged. Firstly, our study's conclusions are limited by the relatively small sample size, which may not fully capture the variability in patient responses to ACEI/ARB discontinuation or continuation. The study also involved a single center with a predominantly homogeneous population, potentially limiting the generalizability of the findings. Secondly, although we employed standardized neuropsychological tests to assess cognitive function, these measures might not capture all aspects of cognitive change post-surgery. Thirdly, our evaluation of cognitive function was limited to the first postoperative day. Future studies should consider including longer follow-up periods, such as at 1 week, one month, or even longer, to more comprehensively assess the long-term effects of ACEI/ARBs on postoperative cognitive function. Fourthly, the study could not be blinded to the patients, as it was not feasible to conceal whether ACEI/ARBs were continued or discontinued preoperatively. Moreover, our study did not include an evaluation of preoperative sleep quality using tools like the Pittsburgh Sleep Quality Index (PSQI), which is a confounding variable for postoperative cognitive assessments. Lastly, the study did not compare the separate effects of ACEIs and ARBs, nor did it include a control group of patients not taking any antihypertensive medication.

In conclusion, preoperative continuation or discontinuation of ACEI/ARBs did not significantly impact postoperative early cognitive function in elderly patients undergoing noncardiac surgery. Patients in the ACEI/ARBs discontinuation group experienced fewer episodes of intraoperative hypotension and less need for vasoactive drugs, despite a higher incidence of preoperative hypertension. Future research should aim to explore the broader implications of ACEI/ARB management on various postoperative outcomes and delve deeper into patient-specific factors that may dictate the optimal perioperative medication strategy.

## Data Availability

The original contributions presented in the study are included in the article/supplementary material, further inquiries can be directed to the corresponding author.
